# Rehabilitation for people wearing offloading devices for diabetes-related foot ulcers: a systematic review and meta-analyses

**DOI:** 10.1186/s13047-023-00614-2

**Published:** 2023-03-25

**Authors:** K. Jones, M. R. Backhouse, J. Bruce

**Affiliations:** 1 Warwick Clinical Trials Unit, Warwick Medical School, University of Warwick, Gibbet Hill, Coventry, CV4 7AL UK; 2 University Hospitals Coventry Warwickshire NHS Trust, Coventry, UK

**Keywords:** Diabetes-related foot ulcer, Offloading device, Rehabilitation

## Abstract

**Background:**

Offloading devices improve healing of diabetes-related foot ulcers (DFUs) but they can limit mobilisation. Rehabilitation during or after removal of these devices may promote physical activity in a population at risk of poor health outcomes for which inactivity is a reversible risk factor.

**Methods:**

This systematic review examined the effectiveness of rehabilitation interventions to promote physical activity during and/or after wearing an offloading device to treat diabetes-related foot ulcers. Searches using MESH terms and free-text combinations: ‘foot ulcer’, ‘diabetic foot’, ‘casts, surgical’, ‘orthotic devices’ were applied to MEDLINE, Embase, The Cochrane Library and clinical trial registers for randomised and observational studies published to September 2022. Methodological quality assessment of included studies was undertaken using the Cochrane Risk of Bias (RoB 2.0) and Risk of Bias In Non-randomised studies of Interventions (ROBINS-I) tools.

**Results:**

Of 3332 records identified, eight studies (441 participants), four clinical trials and four cohort studies, were included. None delivered or tested a structured rehabilitation programme, but all reported physical activity outcomes during or after device use. People wearing non-removable total contact casts were less active than those wearing devices (SMD -0.45; 95% CI − 0.87 to − 0.04; *p* = 0.03; I^2^ 56%; 4 trials). Diabetes-related foot ulcers in people wearing total contact casts were more likely to heal compared to removable devices at 12 weeks (OR 2.69; 95% CI 0.97 to 7.45; *p* = 0.06; I^2^ = 64%; 4 trials) and 20 weeks (OR 2.35; 95% CI 0.95 to 5.82; *p* = 0.07; I^2^ = 65%; 4 trials).

**Conclusions:**

Despite physical activity being low throughout off-loading treatment, no studies have specifically tested rehabilitation. There is a need to investigate the clinical and cost-effectiveness of rehabilitation programmes in this population. High quality trials are needed to provide robust evidence to support to rehabilitation after DFU treatment.

**Supplementary Information:**

The online version contains supplementary material available at 10.1186/s13047-023-00614-2.

## Background

Diabetes-related foot ulcers (DFUs) are associated with poor health outcomes, including increased risk of infection, lower limb amputation and mortality [1, 2]. Recurrence of DFUs is common, with 40% of people re-ulcerating within a year of healing [3]. The annual healthcare cost of managing DFUs is staggering, estimated at between US$9 and US$13 billion in the USA alone [4].

Offloading devices, such as total contact casts and boots, are recommended for the treatment of DFUs by international working groups [5, 6] and the UK National Institute of Health and Care Excellence (NICE) [7]. These devices redistribute pressure away from the ulcer site, aiming to reduce trauma and promote healing. Non-removable knee-high devices are recommended as the first choice of offloading treatment, although removable devices may be equally preferred by both patients and clinicians as they allow greater freedom and mobility [5, 6].

People with DFUs are generally advised to reduce weightbearing activity as much as possible to improve ulcer healing [8, 9]. Knee-high devices reduce the range of motion at the ankle joint which increases the potential for muscle atrophy and bone mass loss over longer periods [10–12]. A recent systematic review (three trials; *n* = 139) concluded there was limited evidence to support non-weight bearing exercise as an intervention to directly improve ulcer healing, although none of the trials reported negative consequences of non-weight bearing exercise [8]. Others have recently challenged conventional advice to limit activity when appropriate offloading footwear is provided, suggesting inactivity may be detrimental to health [9].

Few studies have examined the broader health-related consequences of prolonged immobilisation associated with offloading devices [13]. Given that inactivity and sedentary behaviour are major risk factors for cardiovascular events, frailty, osteoporosis and poor health-related quality of life, it is important to consider the wider impact of wearing offloading devices [5, 13]. Furthermore, there is limited guidance for healthcare practitioners on how best to reintroduce physical activity and rehabilitate patients, to support and encourage mobility after removal of offloading devices.

The World Health Organization (WHO) defines rehabilitation as a set of interventions designed to optimise functioning and reduce disability [14]. Rehabilitation programmes generally include physical and behavioural components to address impairments associated with acute and chronic health problems [14]. Given that low physical activity can be exacerbated by wearing offloading devices and that exercise should be encouraged in people with diabetes, this review aimed to investigate evidence for rehabilitation of people using offloading devices for DFUs. Although several recent studies have narratively described advances and challenges in offloading DFUs, including impact upon physical activity [13, 15], none of these scoping reviews have systematically examined whether rehabilitation can promote physical activity in people using offloading devices.

This review systematically evaluated the clinical effectiveness of rehabilitation interventions designed to promote or support physical activity in people using offloading devices for DFUs.

## Methods

This systematic review was prospectively registered with the International Prospective Register of Systematic Reviews (PROSPERO CRD42021295178). The Cochrane Handbook for Systematic Reviews of Interventions [16] was followed to guide the systematic approach to article identification, data extraction, risk of bias assessment and data analysis. Review findings in accordance with the Preferred Reporting Items for Systematic Reviews and Meta-Analyses (PRISMA) guidelines [17].

### Search strategy

Searches were undertaken on MEDLINE, EMBASE, The Cochrane Library and clinical trial registers from inception to 1st September 2022. Medical subject headings and free-text combinations included, but were not restricted to: ‘foot ulcer’, ‘diabetic foot’, ‘casts’, ‘surgical’, ‘braces’, ‘shoes’, ‘boots’, ‘footwear’, ‘orthotic devices’ (full search strategy in Supplementary file 1). Search strategies were piloted and iteratively tested across different databases. Rehabilitation-related terms were removed to improve search sensitivity. Reference lists were searched manually and checked for citations of studies fulfilling eligibility criteria. No restriction on language was applied although only English language articles were included due to lack of translation facilities.

### Study selection

Duplicates were removed using Rayyan software [18]. Two review authors independently screened titles and abstracts before undertaking full text screening (KJ and MB/JB). Discrepancies were discussed with the third author and resolved by consensus. Reviewers were unblinded to study authors, institution, and journal.

### Eligibility criteria

Any study delivering any type of structured or unstructured rehabilitation, using the WHO definition of ‘any intervention designed to optimise functioning and reduce disability’ were included [14]. Any supportive intervention targeting physical activity or behaviour to encourage mobility, delivered by trained healthcare professionals, either in the hospital or community setting were included. We accepted ‘activity’ using the WHO definition of physical activity defined as “any bodily movement produced by skeletal muscles that requires energy expenditure” [19, 20].

Eligible programmes were those prescribed or delivered during or after offloading boot or cast treatment in people with DFUs. The primary aim was to investigate the clinical effectiveness of rehabilitation tested within clinical trials. Secondary objectives were to narratively describe the components of any rehabilitation delivered in clinical practice. If no rehabilitation programmes were found, this review aimed to extract and summarise physical activity outcomes from the studies identified. Thus, searches were sensitive rather than specific, to include any experimental and observational study designs (randomised controlled trials, quasi-randomised, uncontrolled before and after studies, systematic reviews, case-control, cohorts, cross-sectional or case-series). No restrictions were placed on type of rehabilitation, study setting, participant age or gender or offloading device. Outcomes included self-report or objective measures of physical activity, (i.e. number of steps/distance mobilised, metabolic equivalents (METS), self-reported activity captured in clinic, by telephone, or questionnaire etc.), adherence to prescribed programme, DFU healing (time, area or occurrence) and any health-related or disease-specific quality of life. Studies were excluded if they were published in languages other than English. Conference abstracts or unpublished data were included if methodological descriptions were provided or where data could be supplemented after contact with study authors.

### Data extraction

A modified Cochrane data extraction form was pilot tested and implemented to record: (a) general study information including country, authors, setting and funding; (b) study characteristics including aim, design, randomisation and unit of allocation (trials only), (c) inclusion/exclusion criteria, participant characteristics including sample size, age, gender, DFU definition and severity (Supplementary file 2), duration of diabetes, type of offloading device; (d) content of rehabilitation intervention, any comparison/control group(s); (e) outcome measures including definitions, assessment time points, unit of measurement, (f) loss to follow-up and study findings. Two review authors independently extracted data, with disagreements resolved through consensus of a third review author.

### Risk of Bias

The Cochrane Risk of Bias tool (v2.0) [21] was used to assess methodological rigour in clinical trials and the Risk of Bias In Non-randomised studies of Interventions (ROBINS-I) [22] for observational studies. The Cochrane tool considers domains of randomisation, deviations from intended interventions, missing outcome data, measurement of the outcome and selective outcome reporting (graded as low, some concerns or high risk of bias). The ROBINS-I considers domains of confounding, participant selection, classification of interventions, deviations from intended intervention(s), missing data, measurement of outcomes and reported results (graded as low, moderate, no information, serious or critical risk).

### Data analysis

Change data or between-group mean differences (MD) in physical activity, ulcer size and health-related outcomes by treatment group over time is reported. Proportion of ulcers healed by intervention group was compared using odds ratios (ORs) and 95% confidence intervals (95% CI). Objective measures of physical activity, including accelerometer data e.g. steps are described*.* It was planned to undertake either fixed or random effects meta-analyses based upon heterogeneity (I^2^) findings and a random-effects model was used where statistical heterogeneity was noted (I^2^ > 40%). Sensitivity analysis to exclude studies at high risk of bias was performed if substantial heterogeneity (I^2^ > 75%) was detected. Where studies used a different assessment tool to measure the same outcome, the standardised mean difference (SMD) was calculated. For studies that reported multiple follow-up timepoints, data for the intervention duration were reported and final time point data were reported in supplementary materials. To manage multi-arm parallel-group trials that compared continuous outcomes of two active arms against a comparator arm, data were combined into one active arm to allow meta-analyses.

## Results

**Fig. 1:**
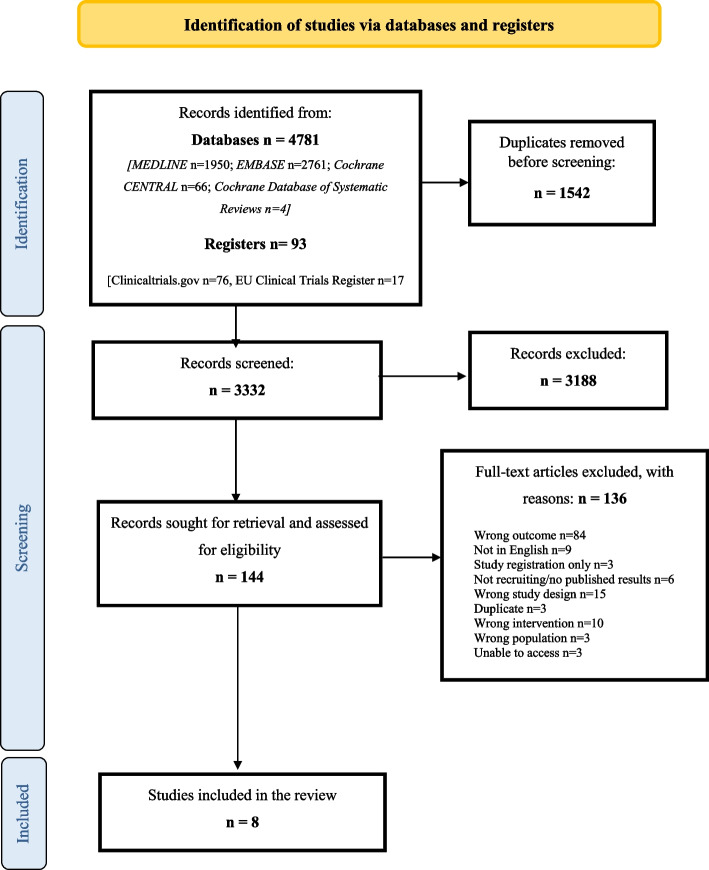
PRISMA flow diagram of literature search and phases of study selection; n, number; CENTRAL, Cochrane Central Register of Controlled Trials

The electronic searching identified 3332 eligible articles. From these, 144 full articles were retrieved for full-text screening. After review, eight studies were included and 136 were excluded for reasons reported in Fig. 1.

### Characteristics of included studies

Of the eight included studies (*n* = 441 participants), four were randomised controlled trials (RCTs) [23–26] and four were prospective cohorts [27–30], reported across multiple publications between 2001 to 2021. Total study sample sizes ranged from 20 to 79 participants; for the RCTs, intervention arms ranged from 18 to 27 participants. Six studies recruited from clinical centres in the USA [23, 24, 27, 28] and of these, two also recruited participants from the UK [29] and Qatar [25]. One European trial recruited from diabetes-related foot clinics in public hospitals in the Netherlands and Germany [26], and one multicentre cohort study recruited from three diabetes-related foot ulcer clinics in Jordan [30].

### Participant characteristics

An overview of RCTs and observational studies is presented in Tables 1 and 2 respectively. Most study recruits were male (333/441; 76%). Mean participant age, where reported, ranged from 53 to 64 years. Two clinical trials failed to describe age [23, 24] and six studies did not report ethnicity/race [23, 25, 27–30]. Of two studies reporting ethnicity, most recruited participants were White Caucasian (84/133, 63%), Hispanic (43/133, 32%), African American (4/133, 3%) or not described (2/133, 2%) [24, 26]. Six studies reported ulcer severity, recruiting participants with a University of Texas Ulcer classification of 1A [23, 27], 1A or 2A [24, 26], 1A up to 2D [29] or Wagner grade-1 classification [28]. One trial did not report foot ulcer severity [25] and the Jordanian cohort study only described ulcer severity in a subset of participants [30].

**Table 1:** Characteristics of Included Cohort Studies

Study	Participants	DFU	Aim	Interventions	Activity Monitor	Follow Up	Activity Outcomes	Other Outcomes	Findings
Ababneh et al. [28] Jordan	*N* = 57 M *n* = 45 F *n* = 12 Mean age 56 (SD 10)	Forefoot or midfoot plantar DFU	To investigate levels and factors/ psychosocial factors, associated with adherence to RCW	RCW	i) Wrist band activity monitor (Fitbit Flex) ii) Boot worn activity monitor Data downloaded after 7 days.	1 week	Total steps/day Activity units = correlated at least 50% of time	Device adherence	Activity Total median 2758 (IQR 1729–4726) daily steps RCW device worn 34% (SD 17%) of total weight-bearing activity. Ulcer healing Not reported
Armstrong et al. [25] USA	*N* = 20 M *n* = 14 F *n* = 6 Mean age 65 (SD 7.6)	Non-infected, non-ischaemic superficial Texas grade 1A.	To evaluate activity and adherence to RCW	RCW	i) Waist worn accelerometer/pedometer (Biotrainer Pro; IM Systems, Boston, MA). ii) Boot-worn accelerometer /pedometer (Biotrainer Pro; IM Systems, Boston, MA). Data recorded 24 hours/day	1 week	Total activity (steps/day). ‘Active use’ = correlated between devices.	Device adherence	Activity Total mean 1219 (SD 821) steps/ day. RCW off 873.7 (SD 828) RCW on 345.3 (SD 219); *p* = 0.01 28% of total daily activity occurred while RCW worn. Ulcer healing Not reported
Saltzman et al. [26] USA	*N* = 40 M *n* = 25 F *n* = 15 Median age 52	Active Wagner Grade I plantar DFU	To evaluate compliance with recommended non-weight-bearing and rate of ulcer healing	TCC	StepWatch monitor (Cyma, Seattle, Washington) incorporated into TCC. Data capture during device change every 1 to 2 weeks	13 weeks	Steps per 24, 48 hours and over remaining 13 weeks.	Ulcer healing by 13 weeks	Activity 24 hours: median 523 steps 48 hours: median 808 steps Median 2083 steps over remaining weeks Ulcer healing 32/40 (80%) healed (median 6 weeks)
Crews et al. [27] USA/UK	N = 79 with adherence data from larger cohort of *n* = 118 *N = 46 UK* *N = 33 USA* M *n* = 66 F *n* = 13 Mean age 56.5 (9.6)	Texas grade 1A up to 2D	To assess association between off-loading adherence and DFU healing (Secondary analysis)	RCW *n* = 61 Sandal *n* = 13 Other *n* = 5	i) Waist worn activity monitor (Lifecorder Plus, Suzuken) ii) Lifecoder Plus (Suzuken) device concealed in off-loading device. Data uploaded by clinician. Adherence = boot device correlated > 50% of waist device activity.	6 weeks	Physical activity (hours/day)	Change in ulcer area; Device adherence (days); *Not reported* Hospital Anxiety Depression Scale; NeuroQOL; IPQ-R.	Activity Mean hours/day active 6.7 (SD 3.8) Device adherence mean 35 (SD 10) days or 59% of time active. Ulcer Healing 19/79 (24%) Mean ulcer size reduction 230 mm^2^ to 106mm^2^; *p* = 0.001.

*DFU* Diabetic Foot Ulcer, *IQR* Interquartile range, *TCC* Total Contact Cast; *RCW* Removable Cast Walker, *IPQ-R* Revised Illness Perception Questionnaire, *NeuroQoL* Neuropathy and Foot Ulcer Quality of life

**Table 2:** Characteristics of included randomised controlled studies

Study	Participants	DFU	Aim	Interventions	Activity Monitor	Follow-up	Outcomes Activity	Outcomes Other	Findings
Armstrong et al. [21] USA/UK	*N* = 63 M *n* = 52 F n = 11 Age – NR	Neuropathic Texas grade 1A plantar DFU	To compare effectiveness of TCCs, RCWs and half-shoes on neuropathic DFU healing	Total contact cast (TCC) *n* = 19 vs RCW *n* = 20 vs Half-shoes *n* = 24	Pedometer (Sportline, Campbell, CA); location not specified Data recorded by study personnel at weekly visits	12 weeks	Total steps	Ulcer healed (Y/N) Time to healing Ulcer area	Activity Daily steps TCC: 600 (SD 320) Half shoe: 1462 (SD 1452) *p* = 0.04 RCW: data not reported. Mean difference (SD) TCC vs RCW = 768 (SD 563); *p* = 0.67 Mean difference (SD) not reported for RCW vs. Half shoe; *p* = 0.15. Ulcer healing: TCC 90%, RCW 65% Half shoe 58%, Other data reported graphically. TCC 90% vs RCW + Half shoe combined 61%; *p* = 0.03.
Lavery et al. [22] USA	*N* = 73 M *n* = 37 F *n* = 36 Age – NR	Texas grade 1A/2A plantar DFU	To evaluate efficacy of TCC, healing sandals and removable boot with shear-reducing walker on DFU healing.	Healing Sandals *n* = 23 vs TCC *n* = 23 Vs Shear-reducing walker *n* = 27	Waist worn pedometer; brand not reported. Daily count recorded in activity book at night.	12 weeks	Total steps	Ulcer healing; Time to healing; Ulcer area; Device adherence (% time each day); Satisfaction with foot care and device; Device comfort; Sleep activity; Likely to wear device again	Activity Mean (SD) steps/day TCC 1447 (SD 1310) HS 4022 (SD 4652) SRW 1404 (SD 1234) HS vs TCC; p = 0.01 HS vs SRW; *p* = 0.007 Ulcer healing HS 10/23 (44%) TCC 16/23 (70%) SRW 6/27 (22%) Time to healing (weeks) HS 8.9 (SD 3.5) TCC 5.4 (SD 2.9) SRW 6.7 (SD 4.3) HS vs TCC *p* < 0.001 SRW vs TCC *p* = 0.22
Najafi et al. [23] USA/Qatar	*N* = 49 M *n* = 45 F *n* = 4 Mean age 53.7	Non-infected, non-ischaemic plantar neuropathic DFU	To report patterns of physical activity as a function of removable and irremovable offloading modality in people with DFU	RCW *n* = 26 vs Instant TCC *n* = 23	Wearable sensor (PAMSys™, BioSensics LLC, MA, USA) incorporated in a shirt (PAMShirt™). Worn for 48 hours at baseline and once weekly for 48 hours until 12 weeks.	12 weeks	Posture (sitting, standing, lying, walking) as % of total activity; Number of steps; Episodes walking; Gait speed; longest unbroken episode of steps; postural transitions per day (sit-to-stand/ stand-to-sit)	Ulcer healing Ulcer area Time to healing Assessed weekly HbA1C	Activity N steps/day at 4 weeks TCC 2994 (SD 2551) RCW 5902 (SD 3090); *p* = 0.07% time walking: TCC 2.4% (SD 2.1) RCW 4.8% (SD 2.1) *p* = 0.05 Ulcer healing TCC 16/23 (70%) RCW 10/26 (40%) *p* = 0.05)
Bus et al [24] Netherlands/Germany	*N* = 60 M *n* = 48 F n = 12 Mean age range 61–64 years	Texas grade 1A/2A ulcer	To assess efficacy of three removable devices on offload and heal neuropathic plantar foot ulcers.	Bivalved TCC (*n* = 20), Cast Shoe (*n* = 20) Forefoot-Offloading Shoe (FOS) (*n* = 20)	Every second patient fitted ankle worn monitor on contralateral limb for 7 days (StepWatch™, Orthocare Innovations LLC, Oklahoma USA).	20 weeks	Daily strides calculated using manufacturer software	Ulcer healing; Time to healing; Ulcer area; Device adherence (< 50% of time worn). Assessed every 2 weeks	Activity Mean (SD) strides/day TCC: 4150 (SD 1626) Cast Shoe: 3514 (SD 1380) FOS: 4447 (SD 3190) *p* = 0.71 Device non-adherence TCC = 17%; Cast Shoe = 5%; FOS = 5%; *p* = 0.27. Ulcer healing TCC: 11/19 (58%) Cast shoe: 12/20 (60%) FOS: 14/17 (70%) *p* = 0.70

*DFU* Diabetic Foot Ulcer, *TCC* Total Contact Cast, *RCW* Removable Cast Walker, *SRW* Shear-Reducing Walker, *FOS* Forefoot-Offloading Shoe, *HbA1c* Haemoglobin A1c

### Offloading device treatment

Total contact casts [23–26, 28], and removable cast walkers [23, 25, 27, 30, 31] were the most popular devices for ulcer management, used in five studies each. Two studies investigated healing sandals [24, 29] and the remaining devices, used in one study each, included a half-shoe [23], shear-reducing walker [24], cast shoe [26], prefabricated forefoot-offloading shoe [26] with some ‘other’ devices not described [29]. One trial compared two different off-loading devices [25]; the remaining three trials each compared three different types of offloading devices.

### Rehabilitation interventions

None of the eight studies delivered any form of structured or unstructured rehabilitation nor prescribed a physical activity intervention at any time during the period that offloading devices were worn. Furthermore, none delivered rehabilitation after offloading device removal to promote activity or support participants with returning to full mobilisation. All included studies used one or more measures of physical activity, captured either as a primary or secondary outcomes during ulcer treatment.

### Overview of reported outcomes

All studies reported one or more physical activity outcomes, measured objectively using waist or ankle-worn pedometers or wearable sensors attached to clothes or embedded within offloading devices (Tables 1 and 2). Activity outcomes were reported as steps over time (hours, days, or weeks), daily stride count (distance of both right and left step), gait speed, percentage of time spent in different postures (sitting, lying, standing, walking) or in postural transition e.g. sit-to-stand and stand-to-sit. Activity monitors were used for different purposes, to capture periods of (in) activity, to determine adherence to wearing prescribed offloading devices [27] or to examine ulcer healing by activity status [28, 29]. Two studies reported activity outcomes only [27, 30]; the remaining six studies also reported ulcer healing or healing-related outcomes, either as proportion healed, change in size or area, and/or time to healing. None measured ulcer recurrence. Adherence to the offloading device was reported by five studies [24, 26, 27, 29, 30]. Other clinical and patient-reported outcomes included: wound/ulcer infection [24], participant satisfaction (foot care; activity levels; daily activities; offloading device comfort and satisfaction; sleep) [24], neuropathy and foot ulcer-related quality of life [29], complication rate [26], shoe peak pressure [26] anxiety/depression [29], and body posture [25]. Outcomes are reported separately by study design.

#### RCTs

##### Physical activity (four trials; *n* = 242 participants)

**Fig. 2:**
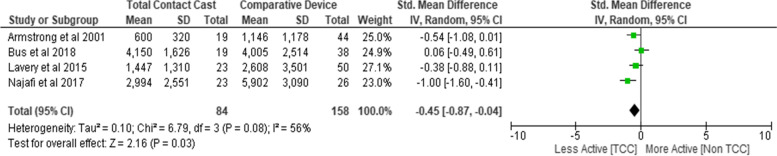
Forest plot comparing physical activity by type of offloading device at 12 weeks. SD, Standard Deviation; Std, Standard; IV, Weight Mean Difference; CI, Confidence Interval; TCC: Total contact cast

All trials reported that participants wearing total contact casts were less active compared to those wearing other removable devices [23–26]. Physical activity after 4 to 12 weeks of ulcer treatment was lower amongst those wearing total contact casts compared to selected other offloading devices (half-shoe, forefoot offloading, healing sandals or removable cast), (SMD -0.45; 95% CI − 0.87 to − 0.04; *p* = 0.03; I^2^ = 56%; four trials, *n* = 242) (Fig. 2). Subgroup analysis of difference in physical activity after wearing a total contact cast versus a removable cast walker (two trials; *n* = 88) demonstrated higher levels of physical activity in those wearing a removable cast walker after 12 weeks (SMD -0.69; 95% CI − 1.32 to − 0.05; p = 0.03; I^2^ = 53%, *n* = 88) (Supplementary file 3).

Najafi et al., [25] reported that walking was very low as a proportion of total daily activity (< 5% of time) – most participants spent their time lying or sitting, regardless of whether they were wearing a total contact cast (91% time sedentary) or a removable cast walker (83% time sedentary). Armstrong et al. [23] found no difference in activity levels between those wearing total contact vs removable casts over 12 weeks but failed to report activity data for all three intervention arms. One trial [26] reported no difference in daily stride count between custom-made offloading devices and a prefabricated forefront offloading shoe, although incomplete data were reported by treatment arm and attrition was high, with only 34/60 (57%) of participants providing activity data at 12 weeks, although authors reported that activity monitors were only given to half the recruited sample.

##### Ulcer healing (four trials, *n* = 242 participants)

**Fig. 3:**
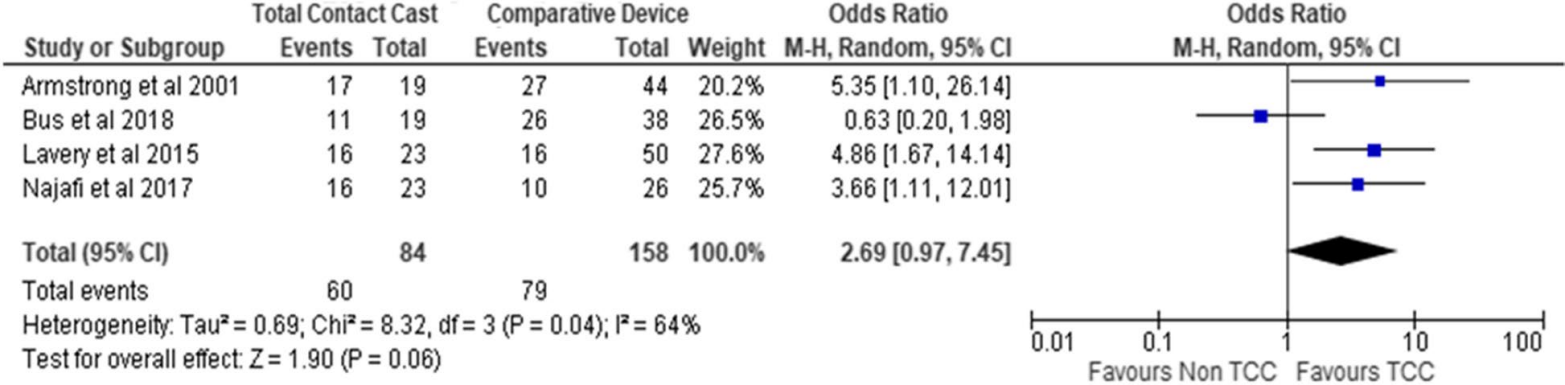
Forest plot of ulcer healing after wearing an offloading device at 12 weeks. TCC, Total Contact Cast; CI, Confidence Interval; M-H, Mantel Haenszel

Overall, odds of ulcer healing were higher in participants wearing non-removable total contact casts compared to all other removable devices combined after 12 weeks of treatment (OR 2.69; 95% CI 0.97 to 7.45; *p* = 0.06; I^2^ = 64%; four trials, *n* = 242) (Fig. 3). Subgroup analysis examining differences in ulcer healing between total contact casts versus removable cast walkers also demonstrated greater odds of healing in those wearing a removable cast walker after 20 weeks (OR 3.93; 95% CI − 1.48 to 10.47; *p* = 0.006; I^2^ = 0%; 2 trials; *n* = 88) (Supplementary file 4). Trial findings of ulcer healing outcomes at 20 weeks by different off-loading devices are reported in Supplementary materials (file 4). One trial [26] reported intention-to-treat and per-protocol data; a in a separate post-hoc sensitivity analyses including only per-protocol findings, this changed the strength but not direction of the estimate of effect at 12 weeks (OR 3.91; 95% CI 2.10 to 7.26; p = < 0.001; I^2^ = 0%; four trials, *n* = 235) (Supplementary file 5) and 20 weeks (OR 3.99; 95% CI 2.11 to 7.57; p = < 0.001; I^2^ = 0%; four trials, *n* = 231 (Supplementary file 6). Although all trials described ulcer size/area at baseline, only two reported area data at follow-up; one trial reported cumulative wound survival graphically (Table 2).

#### Observational studies

##### Physical activity (four studies; *n* = 196 participants)

Four cohort studies measured physical activity for one to 13 weeks after offloading device application. Although one study was described as cross-sectional, recruited participants were monitored for 1 week, it was included as a cohort design [30]. Overall, findings from the observational studies suggested low levels of activity whilst people wore devices. In a cohort with 13 week follow-up [28], 40 participants were advised to be completely non-weight-bearing for the first 48 hours after total contact cast application, although most walked on the cast during this early period (median 808 steps over 48 hours). Total step count was low over the remaining observation period (median total 2083 steps/day). A USA/UK cohort study (*n* = 79) reported higher daily activity levels in those fitted with a removable cast walker, being active for over 6 h per day (mean 6.7 hours; SD 3.8) over 6 weeks, with adherence data suggesting the device was worn over half of time whilst active (59% (SD 22%)) [29]. In contrast, a cohort study with 20 participants found that even when monitored for only 1 week, adherence to a removable cast walker was poor [27]. Participants were less active whilst wearing their cast (mean total daily steps 345 (SD 219) vs 874 (SD 828) when not wearing cast; *p* = 0.01) with only 28% of total daily activity occurring while the cast boot was worn. Similarly, the Jordanian cohort study of 57 participants wearing removable cast walkers also reported poor adherence of 34% of activity time, when monitored for only 1 week [30].

##### Ulcer healing (two studies; *n* = 119 participants)

In the multicentre USA/UK cohort, only 19/79 (24%) ulcers healed over 6 weeks, although mean ulcer size reduced from 230mm^2^ to 106mm^2^ (*p* = 0.001) [29]. Ulcer healing was associated with better adherence to the offloading device although sample sizes were too small for meaningful analyses [29]. Ulcer healing was higher in the cohort study with longer follow-up, with 32/40 (80%) healed after 13 weeks of total contact cast treatment [28].

### Ongoing studies

Registered clinical trials and unpublished studies on the EU Trial and ClinicalTrials.gov registers were searched. Six studies of interest were identified on ClinicalTrials.gov (NCT04280016, NCT04310137, NCT04085926, NCT05236660, NCT04460573, NCT04257565). All are currently open to recruitment with one or more pre-specified activity-related outcomes (Supplementary file 7).

### Risk of methodological bias

Overall methodological quality of the RCTs was poor, with three of four trials being graded at high risk of bias overall and the more recent European trial judged as having some concerns [26] (Supplementary file 8). Only two trials had low risk of bias associated with the randomisation process [25, 26]. Two trials described that outcome assessors or data collectors were blinded to treatment allocation [25, 26] and one trial mentioned blinding but did not describe details [24]. All four trials were at risk of bias in selection of the reported results, due to missing outcome data. Similarly, methodological quality of the observational studies was poor - one was at moderate risk of overall bias [28] and three studies were judged as serious risk of bias [23, 29, 30]. Methodological domains with the least quality concerns related to participant selection and classification of interventions; domains at greatest risk of bias related to outcome measurement, missing data, and bias in selection of reported results (Supplementary file 9). One cohort study aimed to identify factors associated with device adherence in a subset of patients who were already adherent with removable cast walkers for at least 1 month prior to invitation to participate [30].

## Discussion

Diabetes-related foot ulcers are highly prevalent, and their impact is well documented. Although there are myriad potential therapies to encourage ulcer healing, offloading is a central tenet of current management and use of total contact casts or offloading boots is recommended by international guidelines [5–7]. Use of offloading devices is associated with reduced physical activity and an increase in sedentary behaviour [23, 25]. This has been reported in a population in whom activity levels are already known to be significantly lower than activity levels recommended by the WHO, American Diabetes Association and American College of Sports Medicine [19, 32]. Given that inactivity and sedentary behaviour are major risk factors for cardiovascular events, frailty, osteoporosis and poor health-related quality of life [5, 13]; this review sought to systematically review evidence for rehabilitation interventions designed to promote or support physical activity in people prescribed offloading devices. The search strategy was deliberately broad and sensitive to identify any experimental or observational study. Studies specifically evaluating any type of rehabilitation intervention were searched, but also for studies testing alternative offloading devices to investigate whether rehabilitation was prescribed during or after ulcer treatment. The search strategy was revised to remove all MESH and free-text terms relating to rehabilitation and exercise therapy as these were overly restrictive and reduced search sensitivity (to only 91 citations). This involved screening over 5000 citations which maximised inclusivity. Despite the widespread frequency of offloading treatment, no evidence of research evaluating rehabilitation interventions to increase or promote physical activity in people prescribed these devices was found.

Clinical experts have recently challenged traditional wisdom that activity should be limited during DFU offloading, suggesting that prescribed inactivity may be detrimental to health [9]. In addition to the systemic effects of low levels of physical activity, there is growing evidence of the impact of sedentary behaviour on DFUs. Biological tissues respond to the load placed upon them, increasing or decreasing tolerance to stress depending on load [33]. Reduced mechanical loading on the plantar surface of the foot may decrease the ability of tissues to withstand future stress, thereby making tissues more vulnerable to future injury [33]. Recent epidemiological data appears to provide some support for this theory: sedentary time was the strongest predictor of ulcer development and had greater prognostic ability than traditionally recognised risk factors, such as ischaemia and neuropathy [34]. International guidelines now recommend that weight-bearing activity can be carefully encouraged in people with diabetic peripheral neuropathy [5, 13, 32].

Despite the entirely plausible hypothesis that rehabilitation may benefit patients with DFUs, the main finding of this systematic review was that there was no evidence to conclude whether rehabilitation is safe, or clinically or cost-effective. This is a missed opportunity to improve outcomes in people undergoing active ulcer treatment. There is clinical uncertainty and given the scale of the clinical problem, further research is urgently needed to develop and test a targeted rehabilitation package within a high quality, well-designed clinical trial.

Findings are considered in relation to the wider literature. A recent systematic review examined weight-bearing activity amongst people with or at risk of any diabetes-related foot disease. Average mean steps per day was summarised from six studies (*n* = 186 participants; mean steps 4248) but these studies were of people with DFU undergoing any ulcer treatment regime [35]. Jarl et al., [13] investigated weight-bearing activity in those specifically wearing offloading devices, aiming to address clinical uncertainty regarding activity and ulcer healing. This comprehensive, well-written systematic review updated previously reported searches up to January 2020. Results were presented narratively, with inconclusive findings. Quality assessment was not undertaken in either of these recent systematic reviews. Finally, Lazzarini et al. updated a systematic review on offloading interventions focusing on healing outcomes, with ambulatory activity reported as a surrogate outcome. Only two studies [23, 24] contributed to moderate quality evidence statements regarding ambulatory activity (‘*non-removable and removable knee-high offloading devices seem to be associated with similar reductions in ambulatory activity’*) [15].

This systematic review is the first to incorporate meta-analyses, summarising the clinical effectiveness of total contact casting compared to non-total casting on physical activity (step counts) and ulcer healing outcomes. Odds of DFU healing in people using a total contact cast were over twice that compared to removable devices over 3 to 5 months follow-up was found. This finding supports clinical guidelines recommending total contact casts [5–7], although suggests these patients are less active which will inevitably exacerbate cardiovascular risk.

The quality assessment identified various methodological weaknesses in the published research. Common design issues in the clinical trials included small sample sizes (low power), potential biases in the randomisation process, failure to undertake blinded outcome assessment, and lack of intention-to-treat analyses. Moreover, studies often reported multiple outcomes without adjusting statistical analyses for multiplicity. Previous reviews highlight that weight-bearing activity is a secondary or surrogate outcome, rather than the primary aim, limiting interpretation of the relationship with ulcer healing. These methodological issues inevitably influence the certainty of clinical conclusions, but importantly, could be addressed in in future research. These findings concur with previous systematic reviews highlighting the paucity of high-quality research in this area [15].

This systematic review benefits from robust methods in keeping with PRISMA and Cochrane guidelines [17, 36]. Searches were comprehensive, with screening, data extraction and quality appraisal assessments undertaken independently by at least two review authors. Nevertheless, there are some limitations to acknowledge Although an extensive search of major bibliographic databases and trial registers was undertaken, eligibility was restricted to only those studies published in English. All abstracts of non-English studies, where available, were scrutinised but none were considered relevant. As recommended by the Cochrane Collaboration [36], transparency in reporting outcomes that could not be included in meta-analyses were presented narratively. The principal limitation lies in the meta-analysis and stems from the clinical heterogeneity of interventions and outcome measures to determine physical activity. Studies used different parameters to quantify physical activity, collected at variable timepoints and measured over different durations. Although most objective measures were accelerometer-based activity monitors, these were usually poorly validated in the DFU population, and little thought was given to the comparability of devices and outputs. This is an important area in which even basic questions such as ‘what to measure’ and ‘how to measure it’ remain unanswered. These should be addressed before an evidence-based consensus can emerge on the wider and more meaningful use of objective measures of physical activity in people with DFUs.

In summary, this systematic review found no evidence for rehabilitation interventions designed to promote or support physical activity in people using offloading devices for DFUs. This meta-analysis confirmed the clinical effectiveness of total contact casting on ulcer healing, but the quality assessment identified various methodological concerns with included studies. High quality research is needed to address these deficiencies and explore the clinical and cost-effectiveness of rehabilitation in people with DFUs.

## Supplementary Information

**Additional file 1.** Full Search Strategy.

**Additional file 2: Table S1.** The University of Texas Staging System for Diabetic Foot Ulcers (1). **Table S2.** The Wagner Ulcer Grade Classification System (2).

**Additional file 3: Fig. S1.** Forest plot of change in physical activity after wearing an offloading device. SD, Standard Deviation; Std, Standard; IV, Weight Mean Difference; CI, Confidence Interval; TCC, Total Contact Cast.

**Additional file 4: Fig. S2.** Forest plot of ulcer healing using intention to treat data after wearing an offloading device at 20 weeks. TCC, Total Contact Cast; CI, Confidence Interval; M-H, Mantel Haenszel.

**Additional file 5: Fig. S3.** Forest plot of ulcer healing using per-protocol data after wearing an offloading device at 12 weeks. TCC, Total Contact Cast; CI, Confidence Interval; M-H, Mantel Haenszel.

**Additional file 6: Fig. S4.** Forest plot of ulcer healing using per-protocol data after wearing an offloading device at 20 weeks. TCC, Total Contact Cast; CI, Confidence Interval; M-H, Mantel Haenszel.

**Additional file 7: Table S3.** List of ongoing trials of interest open to recruitment.

**Additional file 8: Fig. S5.** Risk of Bias Graph.

**Additional file 9: Fig. S6.** Risk of Bias Graph. **Fig. S7.** Risk of Bias Summary Plot: ROBINS-I Tool.

## Data Availability

All data are available from the corresponding author on reasonable request.

## Abbreviations

CI: Confidence Intervals
DFU: Diabetes-Related Foot Ulcer
MD: Mean Difference
METS: Metabolic Equivalents
NICE: National Institute of Health and Care Excellence
OR: Odds Ratio
PRISMA: Preferred Reporting Items for Systematic Reviews and Meta-Analyses
RCT: Randomised Controlled Trials
ROBINS-I: Risk of Bias In Non-randomised studies of Interventions
SMD: Standardised Mean Difference
WHO: World Health Organization
