# Supplementary material for: Rehabilitation for people wearing offloading devices for diabetes-related foot ulcers: a systematic review and meta-analyses
Source: J Foot Ankle Res. 2023 Mar 25;16:16. doi: 10.1186/s13047-023-00614-2 (PMC10039553; doi:10.1186/s13047-023-00614-2)
Supplement: Supplementary file 1 — Additional file 1. Full Search Strategy. [file 13047_2023_614_MOESM1_ESM.docx]

**Supplementary File 1**

**Full Search Strategy**

**Medline/ EMBASE**

exp foot ulcer/

exp diabetic foot/

(diabet* adj3 ulcer*).tw.

(diabet* adj3 (foot or feet)).tw.

(diabet* adj3 wound*).tw.

(diabet* adj3 defect*).tw.

OR/1-6

exp Shoes/

(shoe$ or boot$ or footwear).mp.

(heel adj elevat$).mp.

(elevat$ adj device$).mp.

(foot adj elevat$).mp.

(foot adj lift$).mp.

(cast or casts or casting).mp.

(aircast$ or scotchcast$ or neofract).mp.

(brace* or bracing).mp.

walker*.mp.

(pressure adj2 device$).mp.

OR/8-18

7 and 19

**Cochrane Library**

MeSH descriptor: [Foot Ulcer] explode all trees

MeSH descriptor: [Diabetic Foot] explode all trees

(diabet* near/3 ulcer*):ti,ab,kw

(diabet* near/3 (foot or feet)):ti,ab,kw

(diabet* near/3 wound*):ti,ab,kw

#6 {OR #1‐#5}

MeSH descriptor: [Casts, Surgical] explode all trees

MeSH descriptor: [Shoes] explode all trees

MeSH descriptor: [Walkers] explode all trees

MeSH descriptor: [Orthotic Devices] explode all trees

Footwear OR shoe* OR insole* OR inlay* OR orthoses
OR orthosis OR orthotic*

cast* OR casting*

brace* OR bracing* OR Walker* OR Removable boot OR prefab*
OR offloading* OR off-loading*

(footwear):ti,ab,kw

orthotic next device*

orthotic next therap*

[OR #7-#16]

#18 [AND #6 and #17]

(pressure next relie*):ti,ab,kw

(pressure next device*):ti,ab,kw

(pressure next redistribution*):ti,ab,kw

((foot or feet) near/2 pressure):ti,ab,kw
((foot or feet) near/2 protect*):ti,ab,kw
((foot or feet) near/2 device*):ti,ab,kw

(remov* near/3 boot):ti,ab,kw

(remov* near/3 cast):ti,ab,kw

(irremov* near/3 boot):ti,ab,kw

(irremov* near/3 cast):ti,ab,kw

(non-remov* near/2 device):ti,ab,kw

#30 [OR #19-#29]

#31 [AND #6 and #30]

#32 [OR #18 and #31]
