# Supplementary material for: Rehabilitation for people wearing offloading devices for diabetes-related foot ulcers: a systematic review and meta-analyses
Source: J Foot Ankle Res. 2023 Mar 25;16:16. doi: 10.1186/s13047-023-00614-2 (PMC10039553; doi:10.1186/s13047-023-00614-2)
Supplement: Supplementary file 2 — Additional file 2: Table S1. The University of Texas Staging System for Diabetic Foot Ulcers (1). Table S2. The Wagner Ulcer Grade Classification System (2). [file 13047_2023_614_MOESM2_ESM.docx]

**Supplementary File 2**

**Table S1.** The University of Texas Staging System for Diabetic Foot Ulcers (1)

| **Stage** | **Grade 0** | **Grade I** | **Grade II** | **Grade III** |
| --- | --- | --- | --- | --- |
| **A** | Pre- or post-ulcerative lesion completely epithelialized | Superficial ulcer, not involving tendon capsule or bone | Ulcer penetrating to tendon or capsule | Ulcer penetrating to bone or joint |
| **B** | Infection | Infection | Infection | Infection |
| **C** | Ischemia | Ischemia | Ischemia | Ischemia |
| **D** | Infection &  Ischemia | Infection &  Ischemia | Infection & Ischemia | Infection & Ischemia |

**Table S2.** The Wagner Ulcer Grade Classification System (2)

| **Grade 0** | No open wounds; Cellulitis of deformity may be present |
| --- | --- |
| **Grade 1** | Superficial wound; Wound may be partial or full thickness |
| **Grade 2** | Ulcer extends to involve such structures as ligaments, tendons, the joint capsule, or deep fascia; No abscess or osteomyelitis present |
| **Grade 3** | Deep ulcer; Demonstrates abscess, osteomyelitis, or joint sepsis |
| **Grade 4** | Localised gangrene of part of the forefoot or heel; Involved areas include part of the forefoot or heel |
| **Grade 5** | Gangrene is extensive and involves the entire foot |

**References**

1. Armstrong DG, Lavery LA, Harkless LB. Validation of a diabetic wound classification system. The contribution of depth, infection, and ischemia to risk of amputation. Diabetes Care 1998;21:855-859

2. Wagner FW, Jr. The diabetic foot. Orthopedics 1987;10:163-172
