# Supplementary figures and images for: Rehabilitation for people wearing offloading devices for diabetes-related foot ulcers: a systematic review and meta-analyses
Source: J Foot Ankle Res. 2023 Mar 25;16:16. doi: 10.1186/s13047-023-00614-2 (PMC10039553; doi:10.1186/s13047-023-00614-2)

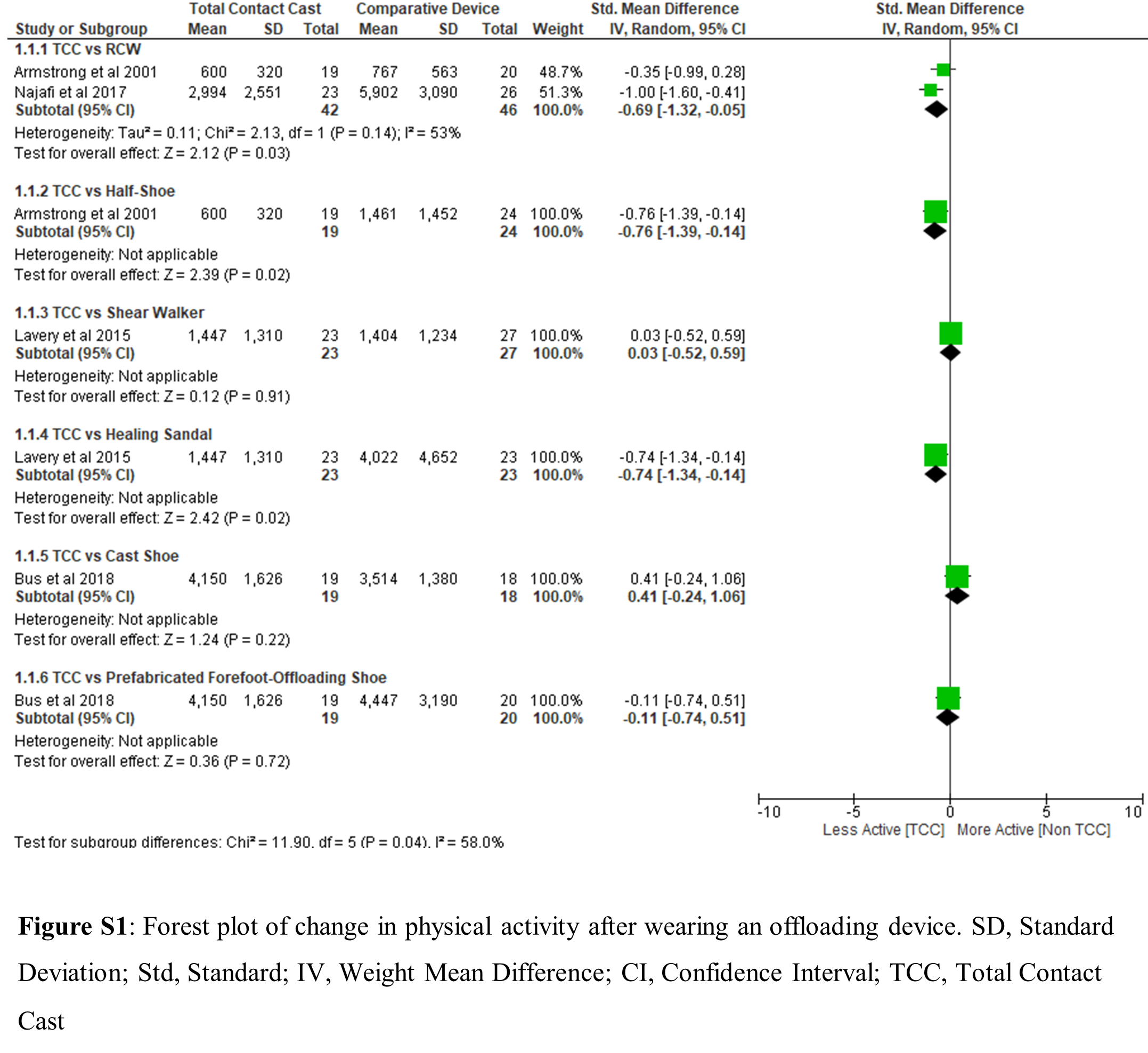

Supplement: Supplementary file 3 — Additional file 3: Fig. S1. Forest plot of change in physical activity after wearing an offloading device. SD, Standard Deviation; Std, Standard; IV, Weight Mean Difference; CI, Confidence Interval; TCC, Total Contact Cast. [file 13047_2023_614_MOESM3_ESM.png]

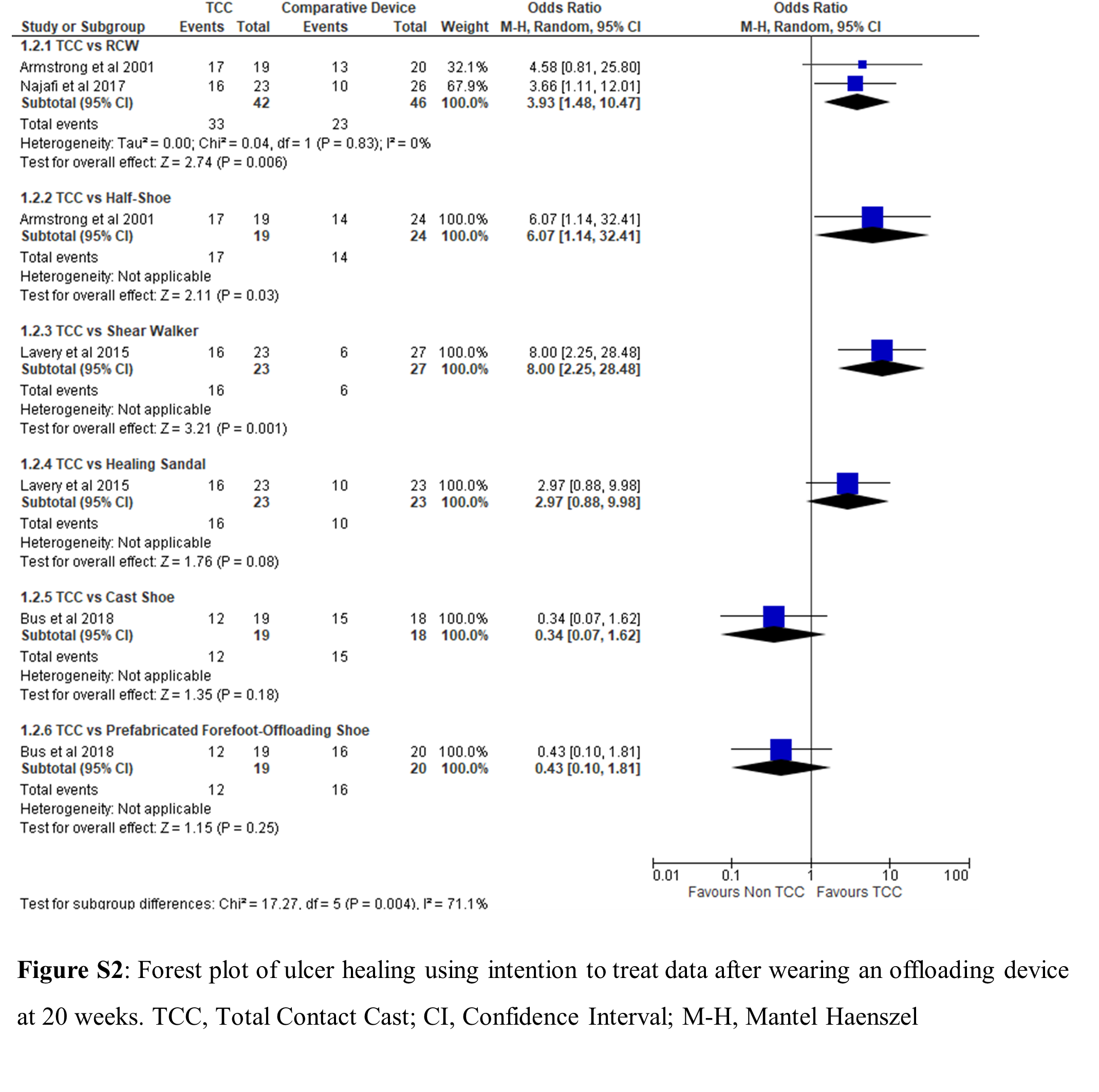

Supplement: Supplementary file 4 — Additional file 4: Fig. S2. Forest plot of ulcer healing using intention to treat data after wearing an offloading device at 20 weeks. TCC, Total Contact Cast; CI, Confidence Interval; M-H, Mantel Haenszel. [file 13047_2023_614_MOESM4_ESM.png]

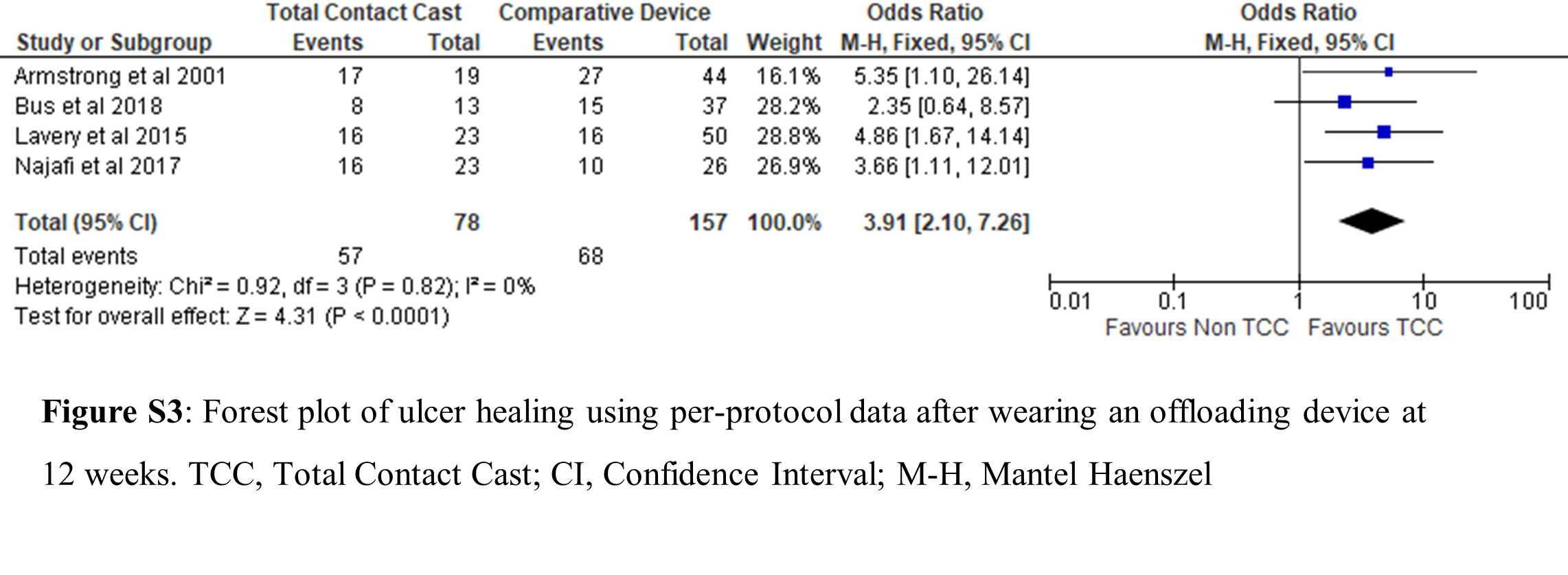

Supplement: Supplementary file 5 — Additional file 5: Fig. S3. Forest plot of ulcer healing using per-protocol data after wearing an offloading device at 12 weeks. TCC, Total Contact Cast; CI, Confidence Interval; M-H, Mantel Haenszel. [file 13047_2023_614_MOESM5_ESM.png]

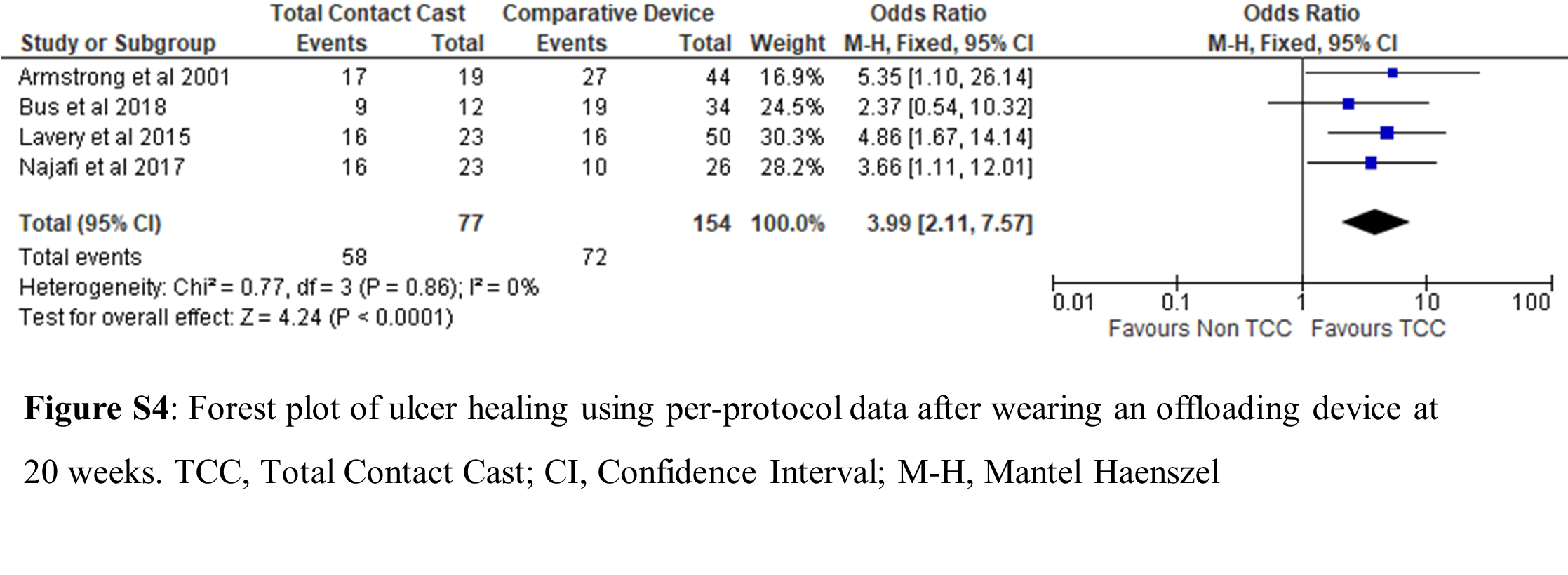

Supplement: Supplementary file 6 — Additional file 6: Fig. S4. Forest plot of ulcer healing using per-protocol data after wearing an offloading device at 20 weeks. TCC, Total Contact Cast; CI, Confidence Interval; M-H, Mantel Haenszel. [file 13047_2023_614_MOESM6_ESM.png]
