# Supplementary material for: Rehabilitation for people wearing offloading devices for diabetes-related foot ulcers: a systematic review and meta-analyses
Source: J Foot Ankle Res. 2023 Mar 25;16:16. doi: 10.1186/s13047-023-00614-2 (PMC10039553; doi:10.1186/s13047-023-00614-2)
Supplement: Supplementary file 7 — Additional file 7: Table S3. List of ongoing trials of interest open to recruitment. [file 13047_2023_614_MOESM7_ESM.docx]

**Supplementary File 7**

**Table S3.** List of ongoing trials of interest open to recruitment

| NCT Number | Status | Study Description | Estimated Completion |
| --- | --- | --- | --- |
| NCT04460573: Smart Boot Use to Measure Offloading Adherence  Country: USA | Recruiting | This study will help people with diabetes who develop neuropathic DFUs. They will compare three different kinds of diabetic boots to see if they can help make it easier for people to wear their boots as instructed. The first group will wear a boot that can't be taken off. The second group will wear a boot that can be taken off, with counselling about how important it is to follow instructions as to how often and when to wear the boot. The third group will wear a "smart" boot, which will interact through a smart watch and smart phone and give them direct feedback re. following their boot wearing prescription. Outcomes include physical activity (walking), sleep and quality of life. | December 2025 |
| NCT05236660: Personalised Assistive Devices Approach for Diabetic Foot Ulcer Prevention (DIASSIST)  Country: Netherlands | Recruiting | Despite availability of various interventions to prevent foot ulcers, recurrence rates remain high. We hypothesise that a multimodal approach incorporating a variety of orthotic interventions that matches an individual person's need can reduce ulcer recurrence with beneficial cost-effectiveness and cost-utility.  Outcomes include cost-utility (primary), adherence to footwear, foot ulcer recurrence over 12 months, quality-adjusted life years. | July 2024 |
| NCT04085926: Sealed Therapeutic Shoe as Treatment of Diabetic Foot Ulcers  Country: Sweden | Recruiting | Total contact casts (TCCs) are effective treatment of plantar DFUs because they offload the ulcer and are non-removable, resulting in high device adherence. However, TCCs are not widely used because they negatively impact gait and daily activities. A new treatment concept was invented, sealed therapeutic shoe, where a shoe with a custom-made insole offloads the ulcer, and the shoe is rendered irremovable to be worn day and night, like a TCC.  In this multicenter RCT, 150 participants will be randomized to TCC or sealed therapeutic shoe.  Outcomes include ulcer healing (primary), skin complications, glycemic control, body mass index, gait function, balance, quality of life, physical activity, and health economics. | December 2027 |
| NCT04310137: Self-directed Versus Therapist-directed Re-loading on Incidence of Ulcer Recurrence  Country: USA | Recruiting | This study will evaluate if how people are told to return to walking after a skin injury affects whether or not they develop new (recurrence) skin breakdown on their feet. The people in this study will have diabetes and have a recently closed foot ulcer. About half will be specifically told how to return to walking and the other half will be told to return to walking slowly. How people naturally return to walking will also be established.  Outcomes: ulcer recurrence, change in walking behaviour (steps/step timing), exercise adherence, biothesiometer, BMI, waist circumference. | December 2023 |
| NCT04280016: The Effect of Exercise on Wound Healing While Off-loading  Country: USA | Recruiting | The purpose of this project is to establish the effect of the addition of exercise to an off-loading intervention on the healing time for people with diabetes mellitus and a foot ulcer.  The benefit of exercise on wound healing in individuals with diabetes has not been elucidated. It is critical to better understand how prescribed exercise effects the healing speed in patients receiving wound care with appropriate off-loading. If exercise does decrease healing time, the benefit to the patient may include lower cost, decreased risk of infection, decreased burden on a caregiver, and improved quality of life.  Outcomes: wound measurement size, participation in exercise, BMI, physical activity data, vibration sense, waist circumference. | May 2023 |
| NCT04257565: Wheeling to Healing: A Novel Method for Improving Healing of Diabetic Foot Ulceration  Country: Canada | Recruiting | The wheeled knee walkers (WKW) is a walking aid that allows total offloading of one foot, requires less physical exertion, and is more stable than crutches or walkers. This research will determine if a WKW would improve wound healing, quality of life, and physical function while decreasing the risk of sound foot ulceration in people with DFU. We will recruit people with DFU under the care of a vascular surgeon, perform standardized baseline measures for each objective, provide a WKW to half of the participants and then re-evaluate all objectives at 6 and 12 weeks.  Outcomes: wound healing, diabetic-related quality of life, risk of ulceration, physical function. | September 2023 |
