# Supplementary material for: Rehabilitation for people wearing offloading devices for diabetes-related foot ulcers: a systematic review and meta-analyses
Source: J Foot Ankle Res. 2023 Mar 25;16:16. doi: 10.1186/s13047-023-00614-2 (PMC10039553; doi:10.1186/s13047-023-00614-2)

**Supplementary file 8**

**Figure S5**: Risk of Bias Graph

RoB 2.0 Tool Domains: 1) Bias arising from the randomisation process; 2) Bias due to deviations from intended interventions; 3) Bias due to missing outcome data; 4) Bias in measurement of the outcome; 5) Bias in selection of the reported result; 6) Overall bias


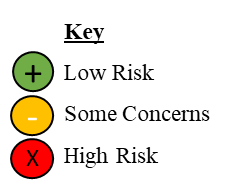

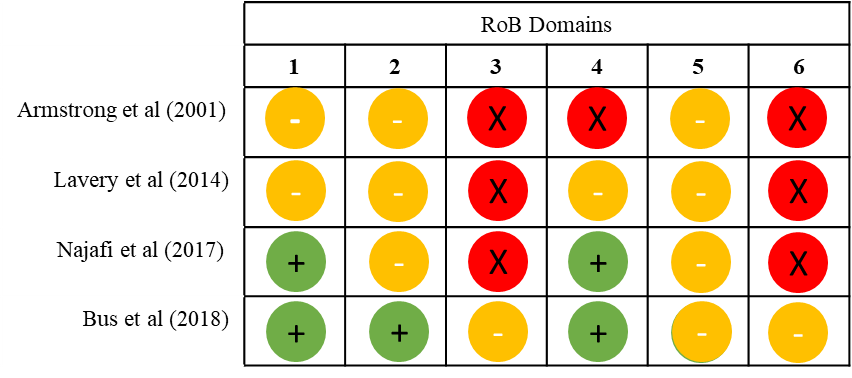


**Figure S6**: Risk of Bias Summary Plot: RoB 2.0 Tool


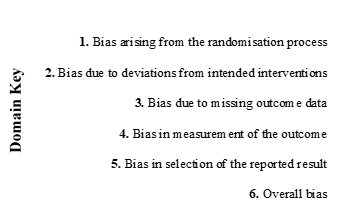

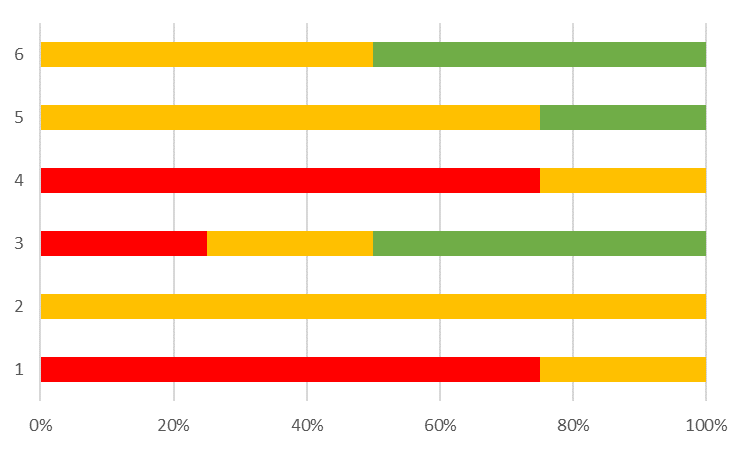

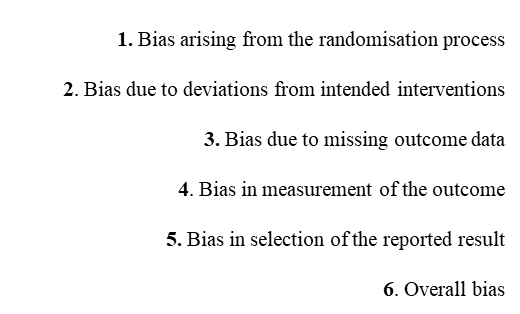

Supplement: Supplementary file 8 — Additional file 8: Fig. S5. Risk of Bias Graph. [file 13047_2023_614_MOESM8_ESM.docx]
