# Supplementary material for: Rehabilitation for people wearing offloading devices for diabetes-related foot ulcers: a systematic review and meta-analyses
Source: J Foot Ankle Res. 2023 Mar 25;16:16. doi: 10.1186/s13047-023-00614-2 (PMC10039553; doi:10.1186/s13047-023-00614-2)

**Supplementary file 9**


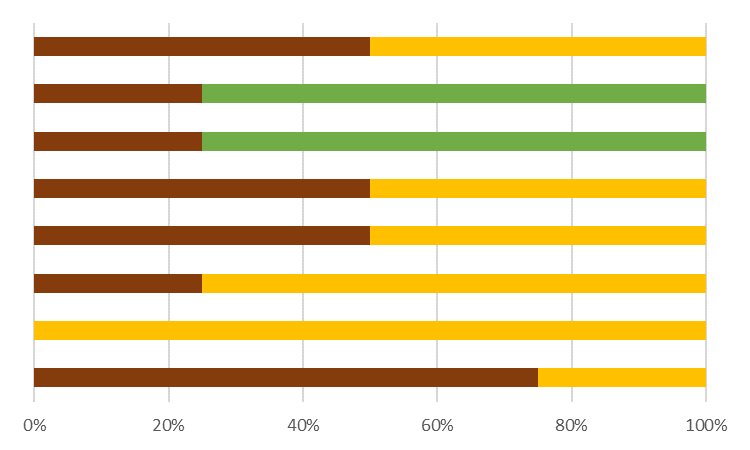

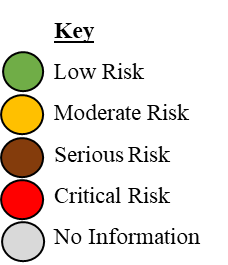


**Figure S6**: Risk of Bias Graph

ROBINS-I Tool Domains: 1) Bias due to confounding; 2) Bias in selection of participants into the study; 3) Bias in classification of interventions; 4) Bias due to deviations from intended interventions; 5) Bias due to missing data; 6) Bias in measurement of outcomes; 7) Bias in selection of the reported result; 8) Overall bias

**Figure S7**: Risk of Bias Summary Plot: ROBINS-I Tool


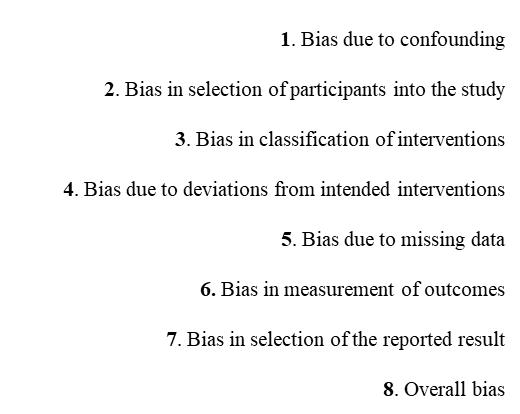

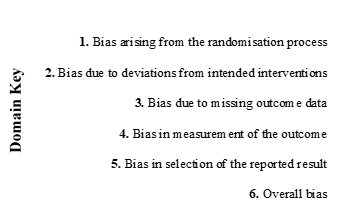

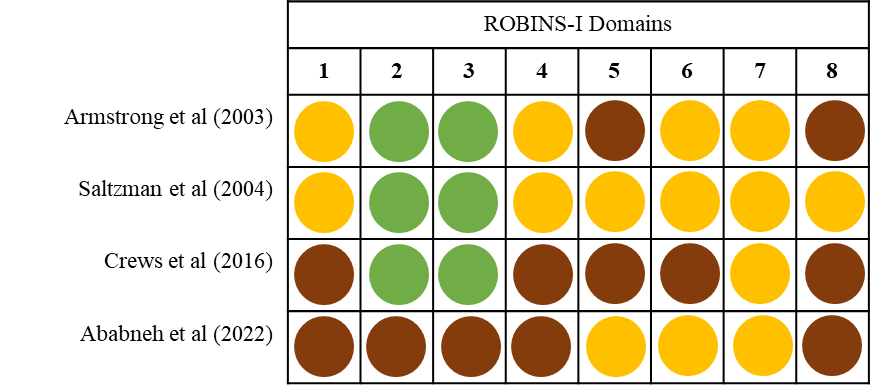

Supplement: Supplementary file 9 — Additional file 9: Fig. S6. Risk of Bias Graph. Fig. S7. Risk of Bias Summary Plot: ROBINS-I Tool. [file 13047_2023_614_MOESM9_ESM.docx]
